# Exophtalmie tardive révélant une fistule carotido-caverneuse post traumatique: à propos d’un cas

**DOI:** 10.11604/pamj.2021.39.153.30184

**Published:** 2021-06-29

**Authors:** Khaoula Boukili, Loubna Elmaaloum, Bouchra Allali, Asmaa Elkettani

**Affiliations:** 1 Service d´Ophtalmologie, Hôpital 20 Août, Université Hassan II, Faculté de Médecine et de Pharmacie, Casablanca, Maroc

**Keywords:** Fistule carotido-caverneuse, artère carotide interne, sinus caverneux, artériographie, à propos d’un cas, Carotid-cavernous fistula, internal carotid artery, cavernous sinus, arteriography, case report

## Abstract

La fistule carotido-caverneuse est une communication anormale entre le système artériel carotidien et le sinus caverneux. L´origine la plus fréquente est congénitale en raison des malformations artério veineuses, mais elle peut être aussi post traumatique. C´est une pathologie rare, pouvant mettre en jeu le pronostic visuel et vital du patient. Nous rapportons ici les cas d´une jeune patiente âgée de 34 ans, qui était victime deux ans avant son admission d´un accident de la voie publique avec impact crânio-facial et au niveau des membres inférieurs, et qui a présenté un an après une exophtalmie gauche d´évolution lentement progressive sans autres signes associés, révélant une fistule carotido-caverneuse gauche. La particularité de notre cas est le délai relativement long entre le traumatisme, l´apparition de l´exophtalmie et le diagnostic de la fistule carotido-caverneuse post-traumatique.

## Abstract (en)

Carotid-cavernous fistula is an abnormal communication between the carotid artery and the cavernous sinus. It is most commonly due to congenital arteriovenous malformations but it can also be caused by a trauma. It is a rare disease which can give rise to visual and life-threatening consequences. We here report the case of a young female patient aged 34 years who had been victim of road accident with craniofacial and lower limb trauma two years before her admission. Left exophthalmos with slowly progressive evolution occurred one year after road accident without other associated signs, revealing left carotid-cavernous fistula. What is peculiar about this case is the relatively long delay between the trauma, the onset of exophthalmos and the diagnosis of post-traumatic carotid-cavernous fistula.

## Introduction

La fistule carotido-caverneuse (FCC) est une fistule directe, à flux élevé, due à une brèche de la carotide interne dans le sinus caverneux. C´est une complication rare mais non exceptionnelle des traumatismes graves cranio-faciaux. Le diagnostic est suspecté cliniquement, il est confirmé par la tomodensitométrie (TDM) ou l´imagerie par résonnance magnétique (IRM) et l´artériographie. La prise en charge thérapeutique a nettement progressé par l´avènement de la neuroradiologie interventionnelle. La chirurgie est indiquée en cas d´échec de la voie endo-vasculaire. L´évolution des symptômes est en général spectaculaire après traitement. Nous rapportons ici les cas d´une jeune patiente âgée de 34 ans, qui a présenté une fistule carotido-caverneuse gauche révélée par une exophtalmie deux ans après un traumatisme crânio-facial.

## Patient et observation

**Information de la patiente**: une patiente âgée de 34 ans, se présente en consultation ophtalmologique pour la prise en charge d´une exophtalmie de l´œil gauche. Elle présentait comme antécédent un accident de la voie publique remontant à deux ans avant, avec un double impact crânio-facial et au niveau des membres inférieurs. La tomodensitométrie cérébrale réalisée initialement n´avait pas objectivé de lésion post traumatique. Par ailleurs elle présentait une fracture de la cheville traitée chirurgicalement. Un an après, la patiente commençait à installer une exophtalmie gauche lentement progressive sans autre signe associé, pour laquelle elle n´avait consulté qu´après presque un an d´évolution.

**Figure 1:**
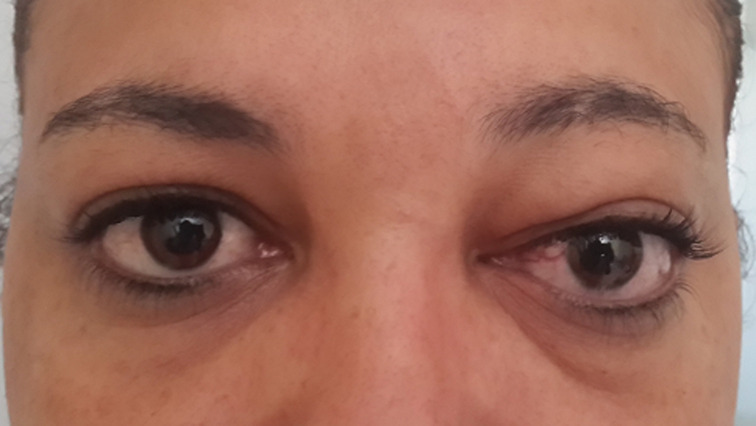
léger ptosis avec une légère exophtalmie axile; on note également une dilatation sinueuse des vaisseaux épiscléraux surtout en nasal

**Résultats cliniques**: l´examen ophtalmologique trouvait : **au niveau de l´œil droit**: acuité visuelle à 110, pas d´anomalie au niveau des annexes, motilité oculaire conservée. **Segment antérieur**: cornée claire, bonne chambre antérieure, bon réflexe photomoteur directe et consensuel, cristallin clair et un bon tonus oculaire. **Fond d´œil**: papille normale avec un bon reflet maculaire. **Au niveau de l´œil gauche**: acuité visuelle à 110. **Au niveau des annexes**: léger ptosis, exophtalmie axile non réductible, non douloureuse et pulsatile, avec une motilité oculaire conservée sans paralysie. On notait également une dilatation sinueuse des vaisseaux épiscléraux surtout en nasal (Figure 1). **Segment antérieur**: cornée claire, bonne chambre antérieure, bon réflexe photomoteur directe et consensuel, cristallin clair et un tonus oculaire élevé à 34mmhg. **Fond d´œil**: excavation papillaire à 4/10 avec une tortuosité des axes veineux. Le reflet maculaire était sans anomalies.

**Figure 2:**
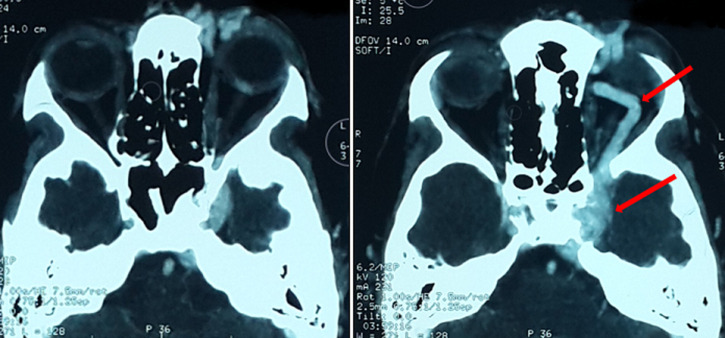
exophtalmie grade 1, avec des signes indirects de fistule carotido-caverneuse: sinus caverneux dilaté et rehaussé en temps artériel et dilatation de la veine ophtalmique supérieure

**Figure 3:**
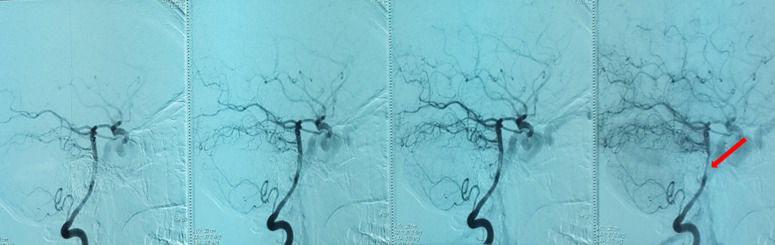
sténose partielle de l’artère carotide interne visible sur l’incidence de profil, zone de disparité du calibre

**Figure 4:**
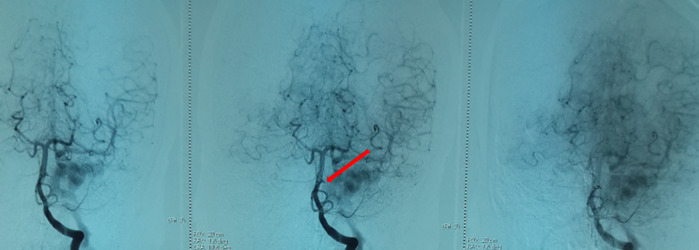
incidence de face visualisant l’anévrysme de 8 mm, image d’addition

**Figure 5:**
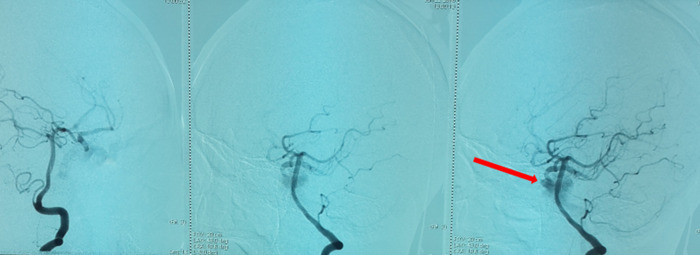
artérialisation du sinus caverneux, il s’opacifie au temps artériel, c’est le signe direct de la fistule carotido-caverneuse

**Démarche diagnostique**: la tomodensitométrie cérébrale demandée en urgence vu le contexte traumatique même ancien avait objectivé une exophtalmie stade I avec un élargissement de la loge caverneuse et son opacification au temps artériel après injection du produit de contraste, et une dilatation de la veine ophtalmique supérieure homolatérale, aspect en faveur d´une fistule carotido-caverneuse gauche (Figure 2). On avait complété par une artériographie qui avait objectivé un aspect en faveur d´une fistule carotido-caverneuse gauche Barrow A, avec un polygone de Willis fonctionnel, et une occlusion de la carotide interne gauche intra-caverneuse avec présence sur sa terminaison d´une image anévrysmale de 8mm intra-caverneuse rompue en plein chenal au sein du sinus caverneux (Figure 3, Figure 4, Figure 5).

**Intervention thérapeutique et suivi**: la patiente était adressée en neuroradiologie pour prise en charge, mais perdue de vue depuis sans bénéficier d´embolisation.

## Discussion

La fistule carotido-caverneuse est une pathologie rare mais non exceptionnelle, l´étiologie traumatique est retrouvée dans presque 75% des cas [1]. Anatomiquement, la partie intra-caverneuse de l´artère carotide interne est le seul système artériel du corps qui traverse un gros plexus veineux. Un traumatisme direct ou indirect de la région crânio-faciale peut entraîner une faiblesse de la paroi musculaire de l´artère carotide interne ou provoquer une véritable lacération produisant un shunt vasculaire à partir d'une artère à haut débit dans un sinus veineux à faible débit, entraînant ainsi la fistule [2].

Sur le plan clinique, les symptômes peuvent apparaitre dans les heures suivant le traumatisme, ou être retardés plusieurs mois après. Ils sont dominés par les signes ophtalmologiques notamment un ptosis, une exophtalmie unilatérale et pulsatile avec un souffle systolodiastolique à l´auscultation de la région périorbitaire et temporale qui disparait à la compression manuelle de l´artère carotide homolatérale au niveau du coup, une diplopie, une ophtalmoplégie, une baisse de l´acuité visuelle, une hypertonie oculaire. On peut avoir aussi des céphalées et des bourdonnements d´oreille. La neuro-imagerie constitue le temps essentiel dans le diagnostic et le traitement des fistules carotido-caverneuses post-traumatiques. La tomodensitométrie quantifie l´exophtalmie en mesurant l´indice oculo-orbitaire, recherche une dilatation de la veine ophtalmique supérieure et recherche un bombement du sinus caverneux, qui constituent les signes indirects de la fistule [3,4]. Elle permet aussi de compléter le bilan lésionnel post-traumatique notamment par la recherche d´une fracture associée. L´imagerie par résonance magnétique a peu d´intérêt puisqu´elle fournit les mêmes renseignements que la tomodensitométrie [5].

Le système de classification universellement adopté dans la littérature pour les fistules carotido-caverneuses est le schéma développé par Barrow et ses collègues [6] : type A: communication directe entre l´artère carotide interne et le sinus caverneux. Type B: fistule durale entre la branche méningée de l´artère carotide interne et le sinus caverneux. Type C: fistule durale entre la branche méningée de l´artère carotide externe et le sinus caverneux. Type D: type B+ type C. A noter que les fistules carotido-caverneuses directes, classés de type Barrow A, sont des shunts à haut débit, et qui se produisent trois fois plus souvent que les types indirects [7]. L´avènement de la neuroradiologie interventionnelle a révolutionné la prise en charge de ce type de fistules. Non traitées, elles engagent le pronostic vital (hémorragies cérébrales ou sous-arachnoïdiennes, épistaxis foudroyant) et fonctionnel du patient (hypertonie oculaire, atrophie optique, baisse de l´acuité visuelle) [8,9]. Le traitement consiste en une embolisation artérielle sélective cérébrale permettant la fermeture de la fistule à l´aide d´un ballonnet interne largable mis en place par voie artérielle en conservant l´axe carotidien interne.

## Conclusion

La fistule carotido-caverneuse post traumatique peut se manifester par une exophtalmie pulsatile plusieurs mois à plusieurs années après le traumatisme, ce qui fait la particularité de notre cas. De ce fait, ce diagnostic doit être évoqué et un bilan neuroradiologique doit être pratiqué quel que soit le délai par rapport au traumatisme.
